# Deep brain stimulation and psychosis as side effect: A case study

**DOI:** 10.1192/j.eurpsy.2024.458

**Published:** 2024-08-27

**Authors:** M. Selakovic, C. Panetsou, T. Karkatsoulis, D. Tsaklakidou

**Affiliations:** Department of Psychiatry, ‘Sismanoglio’ General Hospital, Athens, Greece

## Abstract

**Introduction:**

Deep brain stimulation (DBS) of the subthalamic nucleus (STN) is a therapeutic method used for decades in neurological diseases such as Parkinson’s disease (PD), Huntington’s disease (HD) or dystonia. HD is a rare, inherited, neurodegenerative condition that causes progressive motor deficits, psychiatric symptoms, and cognitive impairment.

**Objectives:**

Moreover, after DBS as a psychiatric side effect has been marked and the etiology of that side effect is not well- understood.

**Methods:**

A case study of a 51 years old male is presented, who developed involuntary movements, for the first time at the age of 17, being diagnosed with Chorea Huntington, was treated with medication without improvement of the symptoms, such as rigidity and bradykinesia. After ten years, based on guidelines, he was treated with DBS, the outcome of which showed complete improvement of neurological symptomatology. Nevertheless, he started to present delusional ideas of reference with his siblings, sleep disturbance, dysphoria and agitation.

**Results:**

Obviously, DBS improved neurological symptomatology permanently. The medical history of our patient has shown the recurrence of psychiatric symptoms as a few mandatory psychiatric hospitalizations and his condition has improved with olanzapine 20 mg/ daily and L.A.I. of paliperidone (once /monthly).

**Conclusions:**

By far, DBS, as a treatment modality, has great potential to modify disease outcomes and potentially cure the devastating genetic neurodegenerative disorder such as chorea. The cases with psychiatric side effects of DBS have been described so rarely, that it’s difficult to formulate conclusions that can be applied to the whole population of patients treated with DBS. In our opinion, in some cases it is possible to effectively treat the psychotic symptoms without resignation from the benefits of DBS.

**Disclosure of Interest:**

None Declared

